# *Cis*-Regulatory Control of the Nuclear Receptor *Coup-TF* Gene in the Sea Urchin *Paracentrotus lividus* Embryo

**DOI:** 10.1371/journal.pone.0109274

**Published:** 2014-11-11

**Authors:** Lamprini G. Kalampoki, Constantin N. Flytzanis

**Affiliations:** Department of Biology, University of Patras, Patras 26504, Greece; Alexander Fleming Biomedical Sciences Research Center, Greece

## Abstract

Coup-TF, an orphan member of the nuclear receptor super family, has a fundamental role in the development of metazoan embryos. The study of the gene's regulatory circuit in the sea urchin embryo will facilitate the placement of this transcription factor in the well-studied embryonic Gene Regulatory Network (GRN). The *Paracentrotus lividus Coup-TF* gene (*PlCoup-TF*) is expressed throughout embryonic development preferentially in the oral ectoderm of the gastrula and the ciliary band of the pluteus stage. Two overlapping λ genomic clones, containing three exons and upstream sequences of *PlCoup-TF*, were isolated from a genomic library. The transcription initiation site was determined and 5′ deletions and individual segments of a 1930 bp upstream region were placed ahead of a GFP reporter cassette and injected into fertilized *P.lividus* eggs. Module *a* (−532 to −232), was necessary and sufficient to confer ciliary band expression to the reporter. Comparison of *P.lividus* and *Strongylocentrotus purpuratus* upstream *Coup-TF* sequences, revealed considerable conservation, but none within module *a*. 5′ and internal deletions into module *a*, defined a smaller region that confers ciliary band specific expression. Putative regulatory *cis*-acting elements (RE1, RE2 and RE3) within module *a*, were specifically bound by proteins in sea urchin embryonic nuclear extracts. Site-specific mutagenesis of these elements resulted in loss of reporter activity (RE1) or ectopic expression (RE2, RE3). It is proposed that sea urchin transcription factors, which bind these three regulatory sites, are necessary for spatial and quantitative regulation of the *PlCoup-TF* gene at pluteus stage sea urchin embryos. These findings lead to the future identification of these factors and to the hierarchical positioning of PlCoup-TF within the embryonic GRN.

## Introduction

Coup-TFs (Chicken ovalbumin upstream promoter-Transcription Factors) are orphan members of the steroid-thyroid-retinoic acid super family of hormone receptors [1], [2]. The structure of Coup-TFs, as for all these receptors, follows a common motif divided into several domains based on specific functional properties [3]. They regulate a plethora of target genes through activation or repression and their ability to heterodimerize with other nuclear receptors to suppress transcriptional activation has been well documented [4], [5].

Coup-TFs are present in all metazoans [6], [7] and show extensive protein sequence conservation across species, which suggests functional similarity [8]. They play a crucial role in homeostasis, organogenesis, neurogenesis and cellular differentiation throughout embryonic development [8]. In vertebrate embryos, Coup-TFs are expressed in the neural ectoderm and mesoderm [9]–[14]. In *D.melanogaster* the Coup-TF ortholog, *svp*, is expressed in the central nervous system and is involved in the differentiation of the photoreceptor cells [2]. In *C.elegans*, *Coup-TF* is a member of the uncoordinated group of genes (*unc-55*) and is involved in motor neuron function and copulation [15], [16]. Knockout and loss or gain of function experiments in vertebrate and invertebrate animals lead to severe abnormalities, mainly in the developing nervous system, and lethality, emphasizing the embryonic significance of Coup-TFs [17]–[19]. Knockdown of PlCoup-TF in sea urchin embryos, via egg injection of morpholino antisense oligonucleotides, results in developmental arrest and inhibition of later stage morphogenesis (unpublished results from this laboratory).

The *S.purpuratus SpCoup-TF* gene [20] is expressed throughout embryonic development [21]. At the gastrula stage, the *SpCoup-TF* gene is expressed in the presumptive oral ectoderm, and at the pluteus stage, mainly in the ciliary band. In the embryonic oral territory, it acts as a repressor of the aboral ectoderm CyIIIb actin gene [22]. The SpCoup-TF protein is maternal and oscillates between the condensed chromatin in mitosis and the nuclear periphery in interface of the early blastomeres, in a cell cycle dependent manner [23]. During larval development the *SpCoup-TF* gene is expressed specifically in neuronal cells (unpublished data from this laboratory). Although Coup-TF's embryonic role has been extensively studied, there is little information about the regulation of this gene in various organisms. To date, there are three known factors involved in the regulation of the *Coup-TFI and Coup-TFII* genes in mice. One of these is Sonic Hedgehog. This protein is secreted from the notochord and is found to play a crucial role in the induction of the chicken and mouse *Coup-TFII* in motor neurons [14], [24]. The transcription factor Ets1 also acts as a positive regulator for the *mCoup-TFI* gene [25]. Retinoids also induce expression of *mCoup-TFI* and *mCoup-TFII* genes *in vitro* [26] and *in vivo* [10], suggesting that Coup-TFs might be direct targets of RA and RX receptors. In higher than physiologic concentrations, retinoic acid has also been shown to act as a ligand of Coup-TFII, which alters its LBD conformation from a repressive to an activating state. These data suggest that Coup-TF may be a ligand regulated nuclear receptor [27].

The herein experiments aim to elucidate the regulation of the *PlCoup-TF* gene in the sea urchin *Paracentrotus lividus* embryo and specifically to determine the regulatory elements that direct *PlCoup-TF* expression in the oral embryonic territory. To this end, we analyzed the *in vivo* function of *PlCoup-TF's* upstream region using the *GFP* gene in a reporter cassette [28], [29]. GFP constructs harboring different deletions and specific mutations of the upstream *PlCoup-TF* region were introduced into sea urchin fertilized eggs via microinjection [30], [31]. Spatial embryonic expression of GFP was monitored by fluorescent microscopy at the pluteus stage, where the definite oral ectoderm and ciliary band, the embryonic territories of endogenous *PLCoup-TF's* expression, are easily discernible. These experiments unveiled an upstream regulatory region (module *a*), which is necessary and sufficient for correct spatial expression of the reporter gene. EMSA experiments, using nuclear extracts from *P.lividus* embryos and three *in silico* identified response elements within module *a*, revealed the presence of transcription factors that specifically bind these elements. Site-specific mutagenesis indicates that correct ciliary band expression of the *PlCoup-TF* gene is mediated by the transcription factors, which bind to the three elements, RE1, RE2 and RE3, within module *a*. Identification of these response elements facilitates the recognition of the corresponding transcription factors, following isolation from embryonic nuclear extracts. This will make feasible the placement of PlCoup-TF downstream of other regulators in the oral ectoderm and ciliary band GRN of the sea urchin embryo. Furthermore, inhibition of PlCoup-TF's expression in the embryo should reveal its downstream gene targets.

## Materials and Methods

### *PlCoup-TF* gene cloning

A pair of primers (*up2: 5′atgttgtggtgcgcaggtcagc* and *do1: 5′gtccggtgtccgcataatgatccgt*) was synthesized based on the known *SpCoup-TF* sequence [20] and used in a PCR reaction with genomic DNA as substrate, isolated from the sperm of an individual *P.lividus* male. The PCR product is a 251 bp fragment within the first exon flanking the AUG codon of the *PlCoup-TF* gene. This fragment was cloned into the pCRII vector (Invitrogen) and sequenced. A *P.lividus* genomic library (a gift from Dr. Valeria Matranga, Palermo, Italy), prepared in the lambda FIXII vector (Invitrogen), was screened using the 251 bp fragment as a hybridization probe. The first round of screening involved 5×10^5^ genomic clones. Isolated positives were subjected to two more rounds of screening from which, two positive overlapping clones were isolated and named “*Α*” and “*Φ*”. The first exon and the upstream region of the gene, contained in the λ clone “*Φ*”, were subcloned and sequenced to facilitate the design of primers and the construction of the GFP expression cassettes.

### Determination of the transcription initiation site

A *PlCoup-TF* gene fragment encompassing 1930 bp upstream and 543 bp 5′UTR region was amplified with PCR, using as substrate the genomic clone “*Φ*” and a pair of primers designed to be complementary to the arm of the phage (*λ*: *5′tctagagagctcgcggcc*) and the 5′UTR (*c*: *5′gatggcgttgagggaatcg*) of the *Pl-Coup-TF* gene. The isolated PCR product was then cloned into the pCRII vector and sequenced. 5′RACE-PCR was performed with the FirstChoice RLM-RACE Kit (Ambion), using 2 µg of polyA^+^ RNA isolated from the ovary of an individual *P.lividus* female. The pair of gene specific primers used for the nested PCR was *tin1 5′cagttctccacgaattgacggc* (in the *PlCoup-TF's* coding region) and *lup3 5′agcggagtaatcgcagctaa* (in the *PlCoup-TF's* 5′UTR). The PCR products were analyzed in a 1,2% agarose gel, purified and cloned into the PCRII vector and sequenced.

### Upstream *PlCoup-TF/GFP* expression constructs

GFP expression constructs included 1930 bp of the upstream region, as well as deletions thereof, and were based on the EpGFPII vector previously used for expression in sea urchin embryos [28]. The first series of constructs use the 1930 bp of the upstream region (from the upstream end of the insert in clone “*Φ*” to +1) fused to *Endo16* gene's basal promoter carried by the vector. The EpGFPII vector also carries part of the actin gene *CyIIa* 5′UTR and its ATG codon. This 1930 bp fragment was amplified with PCR using the primers *LabUpRI*: *5′gacgatgctatcataatagtcatggaattc* and *LabUpBamH1*: *5′ctaggatccgactgatgtttagatggaaag* and the cloned 2.5 kb *PlCoup-TF* fragment as substrate. The *LabUpRI* primer includes an internal EcoRI site (underlined) of the substrate sequence at −1930 and the *LabUpBamH1* primer includes the most upstream transcription initiation site and has a prosthetic BamHI site (underlined) at its 5′ end. The PCR product was digested with the restriction enzymes EcoRI and BamHI and ligated to the EcoRI/BglII digested EpGFPII vector. This construct was designated −1930.

Using the construct −1930 as substrate, six consecutive upstream deletions were amplified with PCR and designated **−1639**, **−1398**, **−781**, **−532**, **−232**, **−19**, respectively. The following pairs of primers were used for each PCR reaction to produce the respective deletions. **−1639**: *Rup7 5′tccttgctgtgagcaattttt*/*GFPpolyAright 5′gtaaaacctctacaaatgtggt*; **−1398**: *Rup6 5′gttttggtatgaagtacgaaacat*/*GFPpolyAright*; **−781**: *Rup2 5′gacccgagtaatcccaacaa*/*GFPpolyAright*; **−532**: *Rup1 5′agcaggacgaaggatttgag/GFPright 5′actgggttgaaggctctcaa*; **−232**: *Rup4 5′ttccggtcttcagaaagttca*/*GFPright*; **−19**: *Rup3 5′ccgtcagaaaaagctttcca*/*GFPright*. Thus, each PCR product contains the respective upstream *PlCoup-TF* fragment fused to the *Endo16* gene's basal promoter and the *GFP* gene. The PCR products were purified, diluted appropriately, mixed with carrier genomic *P.lividus* DNA and used for microinjections.

### Expression GFP constructs with isolated upstream *PlCoup-TF* segments

The 1930 bp upstream region was divided into six sub-regions (**a–f**), which were amplified with PCR and sub-cloned into the EpGFPII vector without the basal *PlCoup-TF* promoter. The recognition sequences for the restriction enzymes EcoRI and BamH1 were added to the 5′ ends of the pairs of primers used for the PCR reactions, respectively. The numbers in parentheses designate the limits of each tested segment. **a (−532/−212)**: *Rup1 5′agcaggacgaaggatttgag*/*Pur4Bam 5′ctaggatcctgaactttctgaagaccggaa*; **b (−781/−513)**: *Rup2 5′gacccgagtaatcccaacaa*/*Pur1Bam 5′ctaggatccactcaaatccttcgtcctgct*; **c (−1079/−762)**: *Rup5 5′tcgaatcacacaccgaaaaa*/*Pur2Bam 5′ctaggatccttgttgggattactcgggtct*; **d (−1398/−1121)**: *Rup6 5′gttttggtatgaagtacgaaacat*/*Pur5Bam 5′ctaggatccttttcggtgtgtgattcgatg*; **e (−1639/−1375)**: *Rup7 5′tccttgctgtgagcaattttt*/*Pur6Bam 5′ctaggatcctttcgtacttcataccaaaac*; **f (−1930/−1681)**: *LabUpRI 5′gacgatgctatcataatagtcatggaattc*/*Pur7Bam 5′ctaggatccaaaaattgctcacagcaagga*. The PCR products were purified and cloned into the PCRII vector. The inserts were then recovered by double digestion with EcoRI/BamH1 from the PCRII vector and ligated to the EcoRI/BglII double digested EpGFPII vector. Plasmid DNAs were linearized with EcoRI, purified, diluted appropriately, mixed with carrier genomic *P.lividus* DNA and used for microinjections.

### Upstream deletions of module *a*

The GFP fusion construct containing module *a*, was used as template to produce with PCR various upstream and internal deletions. For the upstream deletions the following pairs of primers were used (the numbers in parentheses designate the limits of each tested sub-segment). **D-a1 (−473/−212)**: *a1 5′ctaagatcttttccatagaagcctaatccg*/*BglGFPright 5′ctaagatctactgggttgaaggctctcaa*; **D-a2 (−387/−212)**: *a2 5′ctaagatctatagattaatccagaagttgc*/*BglGFPright*; **D-a3 (−334/−212)**: *a3 5′ctaagatctcggagaataacttgtgatgtt*/*BglGFPright*; **D-a4 (−298/−212)**: *a4 5′ctaagatctgtttgagctccgaataccagt*/*BglGFPright*; **D-a5 (−232/−212)**: *a5*: *5′ctaagatcttccggtcttcagaaagttca*/*BglGFPright*. All primers carry a prosthetic BglII recognition site at their 5′ end. PCR fragments were purified, diluted appropriately, mixed with carrier genomic *P.lividus* DNA and used for microinjections.

### Internal deletions into module *a*

Internal deletions into module *a*, were performed with PCR using as substrate the circular plasmid carrying module *a*, and pairs of primers flanking the respective region to be deleted (the numbers in parentheses designate the limits of each deleted internal sub-region). **D1 (−452/−386)**: *a1R 5′ctaagatctcgcattaggcttctatggaaa*/*a2*; **D2 (−386/−333)**: *a2R 5′ctaagatctttcaacaaaatgacacacaat*/*a3*; **D3 (−312/−297)**: *a3R 5′ctaagatctaacatcacaagttattctccg*/*a4*; **D4 (−276/−231)**: *a4R 5′ctaagatctactggtattcggagctcaaac*/*a5*. All primers carry a prosthetic BglII recognition site at their 5′ end. PCR fragments were purified, digested with the restriction enzyme BglII, ligated with T4 DNA ligase and used to transform DH10B bacterial cells. Selected clones were sequenced and used as templates for PCR reactions with the pair of primers *BglRup1* and *BglGFPright*. All PCR products were purified, diluted to the appropriate concentrations, mixed with carrier *P.lividus* genomic DNA and used for microinjections.

### Site specific mutations into module *a*

Site specific mutagenesis was performed by deleting or changing the selected elements with PCR using as substrate the circular plasmid carrying module *a* and pairs of primers flanking the respective site to be deleted. Mutation **−453**: *EtsR 5′ctaagatctattaggcttctatggaaatt* and *EtsF 5′ctaagatcttatacacattaatgactcta*; mutation **−432**: *ApR 5′ctaagatctattaatgtgtatacttcc* and *ApF 5′ctaagatctacaatcaatagaaattataaa*; mutation **−377**: *OtxR 5′ctaagatcttctatttcaacaaaatgacac* and *OtxF* 5′*ctaagatctagaagttgcatgatattgtt*; double mutation **−453/−432**: *EtsR* and *ApF*. All primers contain the BglII restriction site at their 5′ end, which substitutes for the mutated sequence, to enable religation of the amplified plasmids and cloning. Selected clones were sequenced and used as templates for PCR reactions with the pair of primers *BglRup1* and *BglGFPright*. PCR products were purified, diluted to the appropriate concentrations, mixed with carrier *P.lividus* genomic DNA and used for microinjections.

### Collection of animals and embryonic cultures

*P.lividus* individuals were collected from the rocky shores of the Corinth gulf at a depth of 3–5 meters. A specific permission from the Greek authorities for the collection of limited numbers of sea urchins for research purposes was not required. The collection coastal area is not part of a national park or other protected area of the sea. The animals were returned alive to the collection site following the shedding of gametes. The specific location where the animals were collected is: N38.311, E21.783.

Gametes of mature adults were obtained by injection of 0.5 ml 0.5 M KCL into the coelomic cavity. Embryonic cultures for RNA and nuclear protein extract preparations were set up at a maximum concentration of 5×10^6^ embryos per liter of filtered seawater and embryos were let develop at 18°C.

### Microinjection of fertilized eggs

The eggs to be used for microinjection were collected into filtered seawater, de-jellied at pH: 5.5 with the addition of citric acid for 1 min, then the pH was titrated back to 8.3 with the addition of 0.5 M Tris-HCl and filtered through a 65 µm nylon mesh. The eggs were attached onto the covers of 35 mm plastic Petri dishes, pretreated for 2 min with a 2% protamine sulphate solution. Injections were performed using the techniques previously developed [30], [31] with fertilized *P.lividus* eggs. For the 3,317 pluteus stage embryos scored for this study, a total of over 20,000 eggs were microinjected in a period spanning about four years. The duration of the study was due to the short spawning season of *Paracentrotus lividus* in the shallow waters, where the collection of animals took place. In addition, not all batches of eggs give viable or well developing embryos and furthermore, a percentage of embryos do not develop properly due to the microinjection injury. Thus, only well developed plutei from microinjected eggs, were collected and observed for GFP expression. Linear DNAs for microinjection were diluted in 30% glycerol at a concentration of 3–10×10^3^ molecules/pl, plus a five-fold excess of linearized genomic *P.lividus* DNA as carrier. An approximate volume of 3–5 pls was injected into each fertilized egg and embryos were let develop for two days to pluteus stage at 18°C. Epifluorescence observations of developed embryos were carried out with a Zeiss Axioplan microscope using the GFP filter.

### *In silico* upstream promoter analysis

Consensus binding sites for transcription factors within module *a*, were identified using the Transfac MatInspector package (Genomatix). Homologies between upstream *PlCoup-TF* (up to −1930) and *SpCoup-TF* (up to −2500) sequences, were identified using BLAST (NCBI) and the Family Relations II software using a 20 bp window [32].

### Electrophoretic mobility shift assay

Nuclear protein extracts from *P.lividus* post-hatching blastula stage embryos were prepared as described [33]. Double stranded oligonucleotides used as probes, were prepared by annealing the complementary strands of each sequence corresponding to the respective response elements. RE1: *5′cctaatccgggaagtatacaca*; RE2: *5′tatacacattaatgactctacaatca* and RE3: *5′gttgaaatagattaatccagaa*. Binding reactions (15 µl) contained 3×10^4^ cpm of ^32^P end-labeled double stranded oligonucleotide, 1 µg of poly-dA/dT, 1 µg of poly-dI/dC, 20 mM Hepes pH: 7.9, 3 mM MgCl_2_, 1 mM DTT, 50 mM KCl, 8% Glycerol, and 2 µl of nuclear extract. Specific competitors of unlabeled double stranded oligonucleotides, when applicable, were used at 200fold excess to the labeled one. The samples were incubated on ice for 20min and electrophoresed on 6% poly-acrylamide gels in 0,5xTBE buffer at 6°C. Dried gels were exposed to X-ray films.

### WMISH

*In situ* hybridizations to fixed embryos of different developmental stages were performed according to previously published protocols [34], [35]. Digoxygenin labeled sense and antisense hybridization probes (3 ng/ml in hybridization buffer) were prepared by *in vitro* transcription of a PlCoup-TF cDNA clone carrying the entire coding sequence, using T7 and Sp6 RNA polymerases respectively and the *in vitro* transcription kit from Roche.

## Results

### Embryonic expression of the *PlCoup-TF* gene

**Figure 1:**
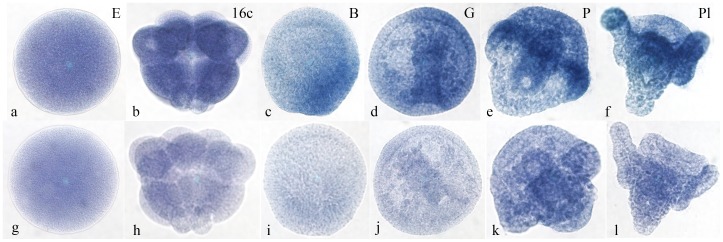
Spatial expression pattern of the *PlCoup-TF* gene. In situ hybridization of *P.lividus* embryos with antisense and sense digoxygenin labeled PlCoup-TF probes. **a–f**: antisense; **g–l**: sense probe hybridization. The maternal PlCoup-TF mRNA seems evenly distributed in the egg and at the 16-cell stage embryo. Zygotic transcripts are expressed in the presumptive oral ectoderm at blastula and gastrula stages and in the ciliary band at prism and pluteus stages. E: Unfertilized egg; 16c: 16-cell stage embryo; B: Hatching blastula; G: Gastrula; P: Prism and Pl: Pluteus.

The expression of the *PlCoup-TF* gene, throughout *P.lividus* embryonic development, was determined by *in situ* hybridization (Figure 1,a-l ). The maternal PlCoup-TF mRNA is detected evenly distributed in the egg (Fig. 1a) and the 16-cell stage (Fig. 1b) embryo. Presumptive zygotic transcripts are detected from the hatching blastula stage (Fig. 1c) onwards. At this, as well as the gastrula stage (Fig. 1d), the *PlCoup-TF* gene is expressed in the cells of the presumptive oral ectoderm, whereas at prism (Fig. 1e) and pluteus (Fig. 1f) stages its transcripts are predominantly detected in the ciliary band. The ciliated cells at the anal side of the band, including the anal arms, seem to be more enriched in PlCoup-TF transcripts than the oral side of the ciliary band. Expression, but to a lesser extent, is also detected at the oral face and the supra-anal ectoderm (Fig. 1e). Sense probe hybridizations result in an even, faint background throughout the embryo and not specific spatial staining (Fig. 1g-l). The presence of PlCoup-TF transcripts in the egg and throughout embryonic development was also determined by quantitative PCR experiments, using total RNAs isolated from Paracentrotus lividus eggs and embryos (not shown).

### Isolation of the *PlCoup-TF* gene

**Figure 2:**
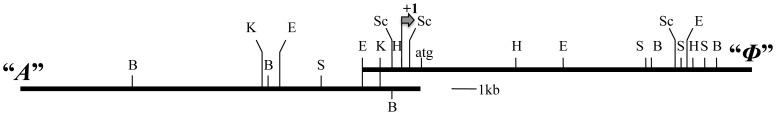
Restriction digest map of the overlapping inserts of genomic clones “*A*” and “*Φ*”. The arrow marked by +1 symbolizes the initiation site and the direction of transcription. ‘atg’, marks the site of translation initiation. The scale (1 kb) is shown by a small bar. The enzymes used for mapping are: B: BamHI; E: EcoRI; H: HindIII; K: KpnI; S: SalI; Sc: SacI.

5×10^5^ clones of a *P.lividus* genomic library were screened with the use of a 251 bp PlCoup-TF specific probe, resulting in the isolation of two positive genomic clones. The two overlapping *P.lividus* λ clones “*Α*” and “*Φ*”, cover about 33.5 kb of genomic region, which encompasses approximately 17 kb of upstream sequences and 16.5 kb of the *PlCoup-TF* gene (Figure 2). The genomic clone “*Φ*”, used in this study, contains 1930 bp of 5′ upstream sequence, 543 bp of 5′UTR sequence and 3 exons, the precise positions of which are not known. The fourth and last exon of the gene, bearing the C-terminal domain of the protein and the 3′UTR are not included in the cloned region contained in the phage “*Φ*”.

### The *PlCoup-TF* gene has multiple transcription initiation sites

**Figure 3:**
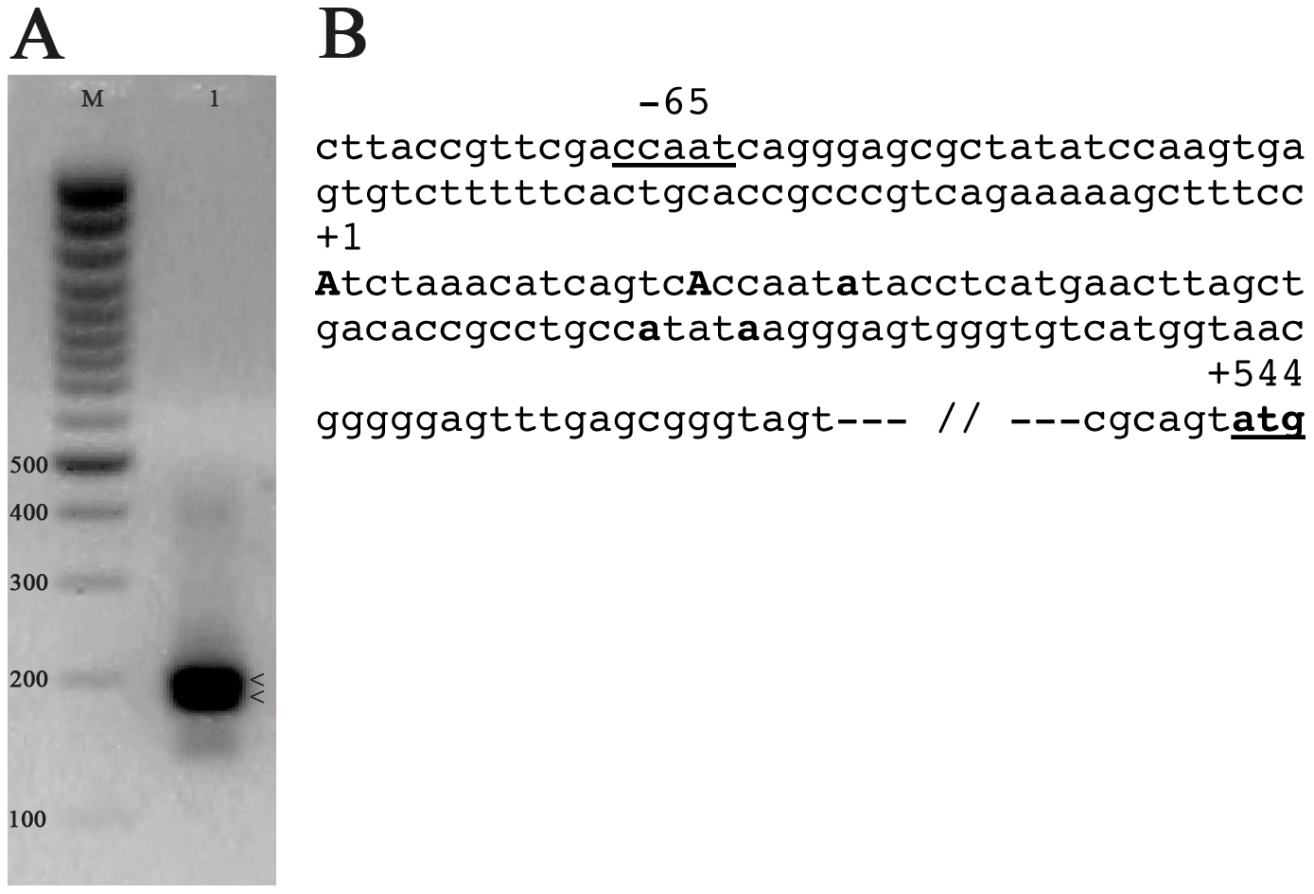
Identification of *PlCoup-TF* transcription initiation sites. **A**: Electrophoretic analysis of the 5'-RACE products. The two arrows point to the major DNA bands produced by the nested PCR (lane 1). The 100 bp ladder (NEB) was used as DNA length reference (lane M). **B**: Sequence of the proximal PlCoup-TF promoter and the positions of the multiple transcription initiation sites (capital and lower case bold characters). The most upstream site was designated as +1. A putative CCAAT box is underlined at position −65. The translation initiation site is located at +544.

Electrophoresis of the nested PCR products indicated two major DNA bands with approximate length of 200 bp (Figure 3A). Sequencing of cloned PCR fragments yielded thirteen isolated clones identifying at least five different transcription initiation sites. The two most upstream sites (at +1 and +16) were represented more frequently by four and five clones respectively (bold As in figure 3B) and correspond to the two major 5′-RACE products observed after electrophoresis. Four additional clones revealed initiation sites at +22 (two clones), +54 (one clone) and +58 (one clone), marked with lower case bold characters in figure 3B. Considering these results the most upstream of the transcription initiation sites was designated as +1 for the herein conducted experiments. An obvious ‘TATA’ element is missing in the proximal upstream sequence, although a putative ‘CCAAT’ box is underlined at −65.

### Upstream deletion analysis of the *PlCoup-TF* gene

**Figure 4:**
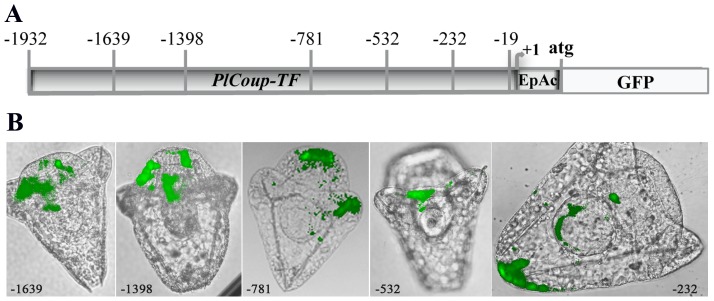
Spatial expression patterns generated by the upstream deletions of the GFP cassette. **A**: Map of *PlCoup-TF's* upstream sequence (1930 bp) fused to the *EpGFPII* reference gene. The bended arrow marks the transcription initiation site. EpAc refers to *Endo16′s* basal promoter and *CyIIa's* kozak sequence and ATG. The numbers above the map indicate the starting point of each upstream deletion. **B**: Composite pictures (GFP epi-fluorescence over bright field image) of embryos resulting from injection of the corresponding deletions −1639 to −232. Constructs −1639, −1398 and −781 show GFP expression in ciliary band and a few mesenchyme cells. Construct −532 shows GFP expression only in ciliary band and construct −232, in the aboral ectoderm. All embryos were photographed at pluteus stage. A picture of an embryo injected with the construct −19 is not shown, since these embryos never exhibited any GFP expression.

A series of deletions into the 1930 bp upstream region was prepared by PCR using gene specific primers (the border of each deletion is marked with a vertical bar in figure 4A) and a downstream GFP primer. Linear DNAs were injected into fertilized *P.lividus* eggs and developed embryos were scored for fluorescence 2 days post fertilization at pluteus stage (figure 4B). Since embryos developing from injected eggs integrate randomly the exogenous DNA, at various early embryonic cleavage stages, ensuing embryonic lineages are mosaic. Thus, rarely all cells of a certain lineage would express the transgene. Embryos exhibiting only one or two fluorescent cells were not scored as expression positive.

A large percentage of the developed embryos exhibit fluorescence except for deletion *−19*. As this result is expected, since only 19 bp of *PlCoup-TF's* upstream sequence are present in this deletion, it is also indicative of *SpEndo16* basal promoter's lack of activity in the absence of additional *cis* regulatory inputs [36]. All other tested upstream sequences result in high percentage of fluorescent embryos. A significant percentage of injected embryos show fluorescence in a small number of secondary mesenchyme cells (2–3 cells) in addition to other tissues. Such fluorescence, seen at similar percentage levels in all injected groups (Me+, Fig. 4B and Table 1), is not considered to be specific expression of the various deletions, but rather background or “leakiness” of the assay.

**Table 1:** Embryonic cell lineage specific expression of the GFP reference gene for each of the upstream deletions, individual upstream segments and mutations.

	# of embryos	%Fluor.embryos	% Cb	% Cb+	% En+	% Ae+	% Me+
**Deletions**							
**−1639**	151	77	19	39	29	23	51
**−1398**	148	75	40	75	21	17	37
**−781**	107	72	56	74	1	3	44
**−532**	102	77	51	82	6	10	37
**−232**	90	82	32	58	24	22	43
**−19**	58	0	0	0	0	0	0
**Segments**							
**a**	237	87	32	83	25	8	47
**b**	117	16	0	53	0	0	100
**c**	98	34	21	33	12	0	67
**d**	30	0	0	0	0	0	0
**e**	40	0	0	0	0	0	0
**f**	66	0	0	0	0	0	0
**Deletions**							
**D-a1**	199	59	15	68	48	18	44
**D-a2**	127	84	6	69	30	28	68
**D-a3**	171	83	8	31	17	8	81
**D-a4**	129	78	13	40	28	8	69
**D-a5**	130	42	0	22	15	9	83
**Internal Deletions**							
**D1**	141	96	10	76	61	41	54
**D2**	241	79	38	81	36	39	10
**D3**	193	76	21	66	35	16	57
**D4**	47	68	28	56	9	25	53
**Mutants**							
**−453**	204	35	72	92	14	14	3
**−432**	202	87	22	83	61	46	5
**−377**	184	66	43	79	26	36	11
**−453/−432**	105	18	37	47	42	37	0

Cb: Ciliary band; Cb+: Ciliary band and other cell types; En+: Endoderm and other cell types; Ae+: Aboral ectoderm and other cell types; Me+: Mesenchyme and other cell types.

The deletion constructs exhibiting both the highest percentage of embryos expressing GFP in the ciliary band and the lowest percentage of embryos expressing the reference gene in other embryonic territories, i.e. aboral ectoderm and endoderm, are *−781* and *−532*. Thus, in this analysis, the smallest upstream segment that fulfills the above stated criteria is from *−532* to *−232* (Fig. 4B and Table 1).

**Figure 5:**
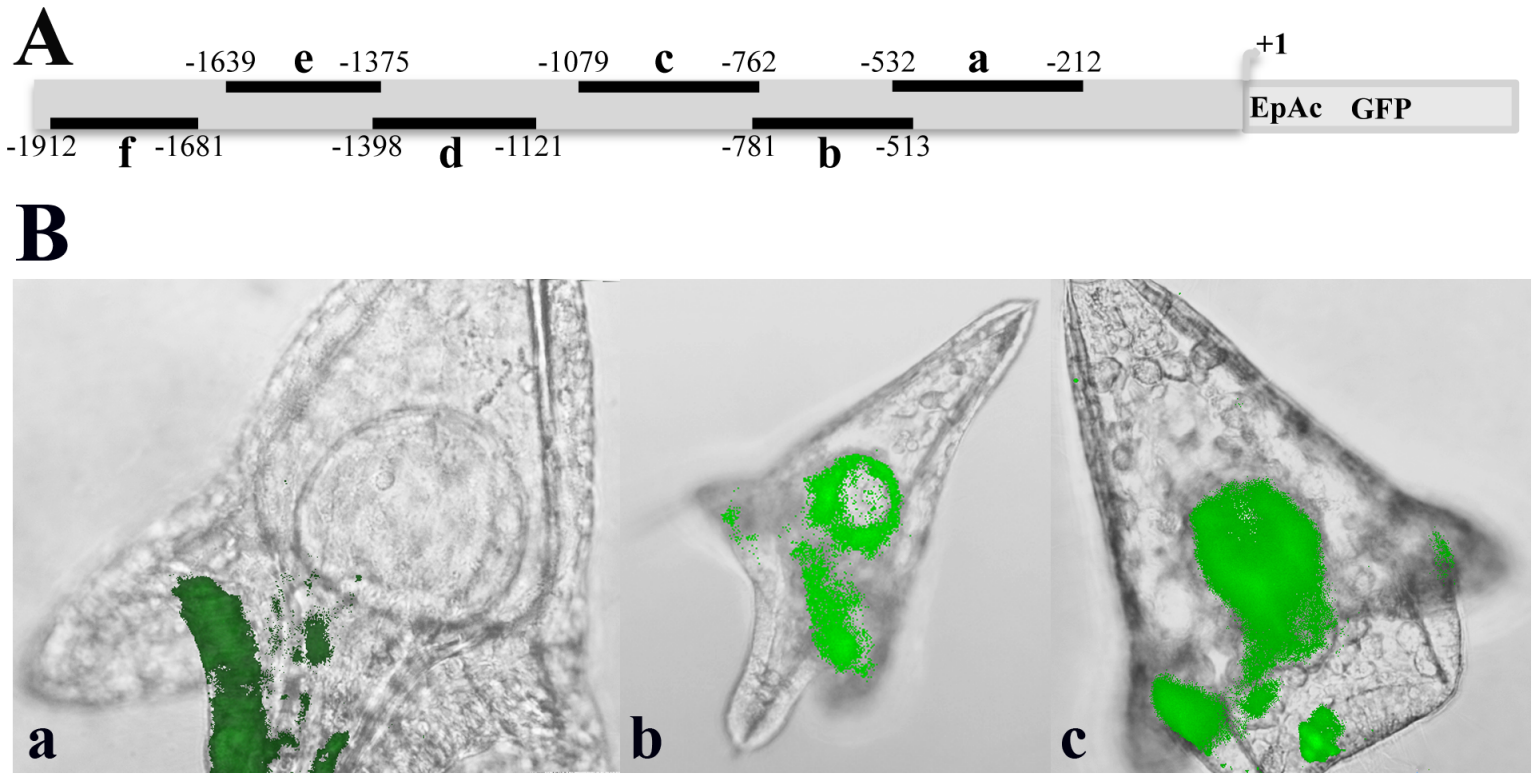
Spatial expression patterns generated by the individual segments a–f fused to the GFP cassette. **A**: Graphical positioning of the upstream *PlCoup-TF* segments (a–f) within the 1932 bp upstream sequence, which were individually fused to the *EpGFPII* reference gene. The numbers surrounding each black bar correspond to the nucleotide borders of each segment. Other designations are as in Figure 4A. **B**: Composite pictures of embryos resulting from injection of segments a–c. Segment a results in GFP expression specifically in the ciliary band, while segments b and c show GFP expression in the ciliary band, endoderm and mesenchyme cells. All embryos were photographed at pluteus stage. Embryos injected with segments d, e and f did not exhibit GFP expression.

Six upstream segments (a–f, Fig. 5A), cloned into the EpGFPII vector, were amplified by PCR as cassettes fused to GFP and injected into fertilized *P.lividus* eggs. Segments d, e and f do not result in any detectable GFP expression. Segment a directs GFP expression mostly in the ciliary band, while b and c show considerably less ciliary band specific expression and much higher non-specific expression in mesenchymal and endodermal cells (Fig. 5B and Table 1). These results agree with the upstream deletion data (Fig. 4), which indicate that the minimal region conferring ciliary band specific expression extends between −532 and −232. Thus, it is evident that segment a, refer to as ‘module *a*’ from hereon, is necessary and sufficient to direct *PlCoup-TF's* specific expression in the ciliary band of the pluteus embryo.

### Comparison of *Coup-TF* upstream regions between *P.lividus* and *S.purpuratus*

**Figure 6:**
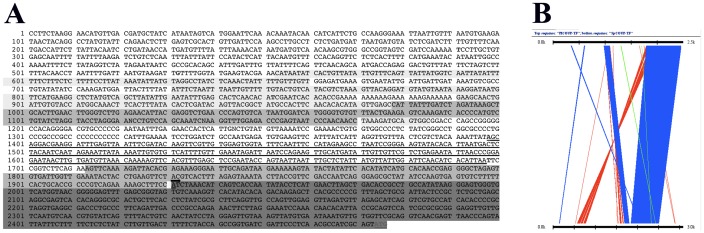
Comparison of *Coup-TF's* upstream and 5′UTR regions between *P.lividus* and *S.purpuratus*. ***A***: The 5′UTR and the upstream sequence of the PlCoup-TF gene, numbered from the −1930 position. The data were obtained by subcloning and sequencing a 2.5 kb fragment of the λ clone “*Φ*” insert (Fig. 2). The shaded areas correspond to various degrees of homology with the corresponding sequence of *SpCoup-TF* as revealed by comparisons using the program BLAST. The lighter the shade, the lesser the homology is between the ortholog genes of the two species. Thus, the darkest shade corresponds to the 5′UTR sequence that exhibits the highest homology. The small black arrow denotes the transcription initiation site. Underlined is the upstream sequence of module *a*, which is not homologous between the two species. **B**: Graphic comparison of the 5′UTR and 5′ upstream sequences of the two orthologous genes, *PlCoup-TF* (top) and *SpCoup-TF* (bottom) using the Family Relations program (32). Crossbars joining the two sequences indicate homology, the thickest of which corresponds to the 5′UTR region.

Using the programs BLAST and Family Relations II, we compared the upstream and the 5′UTR sequences of the orthologous *Coup-TF* genes of *P.lividus* and *S.purpuratus*. We found that the 5′UTR regions (+1 to +543) are extremely conserved (89%) and that the upstream regions of the two genes show some scattered nucleotide homology, which in places is significant. Thus, from −217 to −1 the homology is 76%; from −952 to −745, 77%; from −1201 to −953, 58% and from −1377 to −1270 the homology is 61%. To our surprise, module *a* (underlined in figure 6A), is not conserved between the two species, as shown by both analyses (Fig. 6A and B), although *SpCoup-TF* is also predominantly expressed in the ciliary band at pluteus stage [21].

### Upstream and internal deletions into module *a*

**Figure 7:**
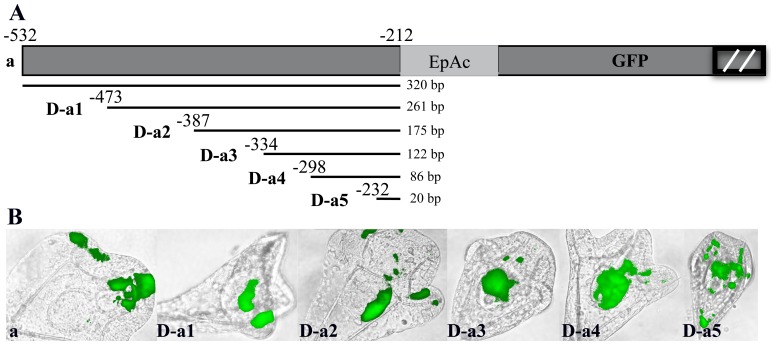
Spatial expression patterns generated by upstream deletions D-a1 to D-a5 of module *a*. **A**: Graphic presentation of the upstream deletions into module a (−532 to −212). Horizontal bars underneath module *a*, represent the size of each deletion (D-a1 to D-a5) and the numbers above them the corresponding upstream border. The numbers at the right of each bar correspond to the size of each fragment tested. EpAc refers to *Endo16′s* promoter and *CyIIa's* kozak sequences as in figure 4A. The two parallel bars within the black box at the right end of the graph denote that the *GFP* gene is not depicted on scale. **B**: Composite pictures of embryos resulting from injection of upstream deletions into module *a*. The entire module *a* shows GFP expression specifically in the ciliary band, while deletions D-a1, D-a2 and D-a3 show expression both in ciliary band and endoderm. Deletions D-a4 and D-a5 show expression in endoderm and mesenchyme cells. D-a5 shows GFP expression in skeletogenic mesenchyme cells (see text for non-specific expression caused by random integration of the GFP cassette). All embryos were photographed at pluteus stage.

To explore the regulative capacity of sub-regions within module *a*, we injected progressive upstream deletions (D-a1 to D-a5), as diagrammed in figure 7A, fused to the reporter gene. Injected sequences differ only in the extent of the upstream *PlCoup-TF* region. By comparison to the entire module *a*, individual deletions resulted in considerable loss of ciliary band specific expression and a parallel increase in ectopic expression (Table 1). Thus, D-a1 exhibits increased expression in the endoderm, suggesting the existence of a negative response element, from −532 to −473, conferring suppression of endoderm expression to the *PlCoup-TF* gene. Deletion D-a2, in addition to higher endoderm expression, results in a 5 fold decreased expression in the ciliary band and an almost 4 fold increased expression in the aboral ectoderm. These results indicate the presence of a positive regulatory element from −473 to −387 regarding ciliary band specific expression, and negative regulatory elements, which suppress expression in aboral ectoderm and to some degree in the endoderm.

A further deletion into module *a*, from −387 to −334 (D-a3), shows a complete loss of spatial specificity (an increase in non specific mesenchyme cell expression), suggesting the existence of perhaps an additional regulatory element within the deleted sequence. Deletions D-a4 and D-a5 show also a high percentage of embryos expressing GFP non-specifically, in mesenchyme cells, and very low levels of other lineage specificity, indicating the absence of regulatory sites from −334 to −212 (Fig. 7B). These results imply that cis-regulatory elements driving PlCoup-TF's expression in the ciliary band, lie in a smaller 200 bp region, between −532 and −334, within module *a*.

**Figure 8:**
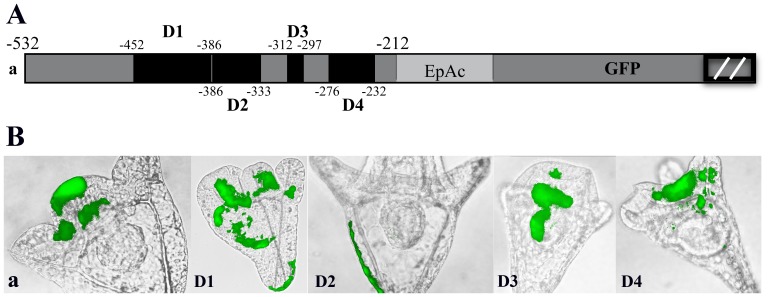
Spatial expression patterns generated internal deletions D1–D4 into module *a*. **A**: Map of the internal deletions. Black boxes correspond to the deleted regions D1–D4. The numbers surrounding each box mark the borders of each deletion. EpAc refers to *Endo16′s* promoter and *CyIIa's* kozak sequences as in figure 4A. The two parallel bars within the black box at the right end of the graph denote that the *GFP* gene is not depicted on scale. **B**: Composite pictures of embryos resulting from injection of the internal deletions D1–D4. The entire module *a* shows GFP expression specifically in the ciliary band, deletion D1 in ciliary band, endoderm and aboral ectoderm and D2 in aboral ectoderm. D3 shows expression in ciliary band and endoderm while D4 shows expression in mesenchyme cells. All embryos were photographed at pluteus stage.

To complement the upstream deletion experiments we performed a series of injections using internal deletions into module *a*, fused to the GFP reporter cassette. Thus, with minor nucleotide differences based on the positions of the primers used, D1 (−452 to −386) corresponds to the region between D-a1 and D-a2, D2 (−386 to −333) to D-a2 and D-a3, D3 (−312 to −297) to D-a3 and D-a4 and D4 (−276 to −232) to deletions D-a4 and D-a5 respectively (figures 8A and 7A). Deletion D1 results in ectopic expression of the reporter, i.e. we observe a three fold decrease in the percentage of embryos exhibiting expression only in the ciliary band and in addition a two and a half fold increase in the endoderm and a five fold increase in the aboral ectoderm (Table 1). Thus, compared to the expression profile of the entire module *a*, deletion D1 exhibits similar ectopic expression as deletions D-a1 and D-a2. It is evident that the sub-region from −452 to −386 contains negative regulatory elements, suppressing the expression of *PlCoup-TF* in the endoderm and the aboral ectoderm territories of the embryo and positive regulatory elements enhancing its expression in the ciliary band (Fig. 8B and Table 1). On the contrary, deletion D2 does not influence ciliary band or endoderm expression, but leads only to ectopic expression in the aboral ectoderm. This result suggests the existence of additional negative regulatory elements between −386 and −333 that suppress PlCoup-TF expression specifically in the aboral ectoderm. The internal deletions D3 and D4, do not exhibit any remarkable differences compared to the expression profile of module *a* (Table 1). Considering the results of the entire deletion analysis of the *PlCoup-TF* upstream sequences, it is apparent that the 120 bp region from −452 to −333 includes the minimal positive and negative regulatory information for the correct spatial expression of the gene in the ciliary band of the pluteus embryo.

### Nuclear factors binding to response elements RE1, RE2 and RE3

**Figure 9:**
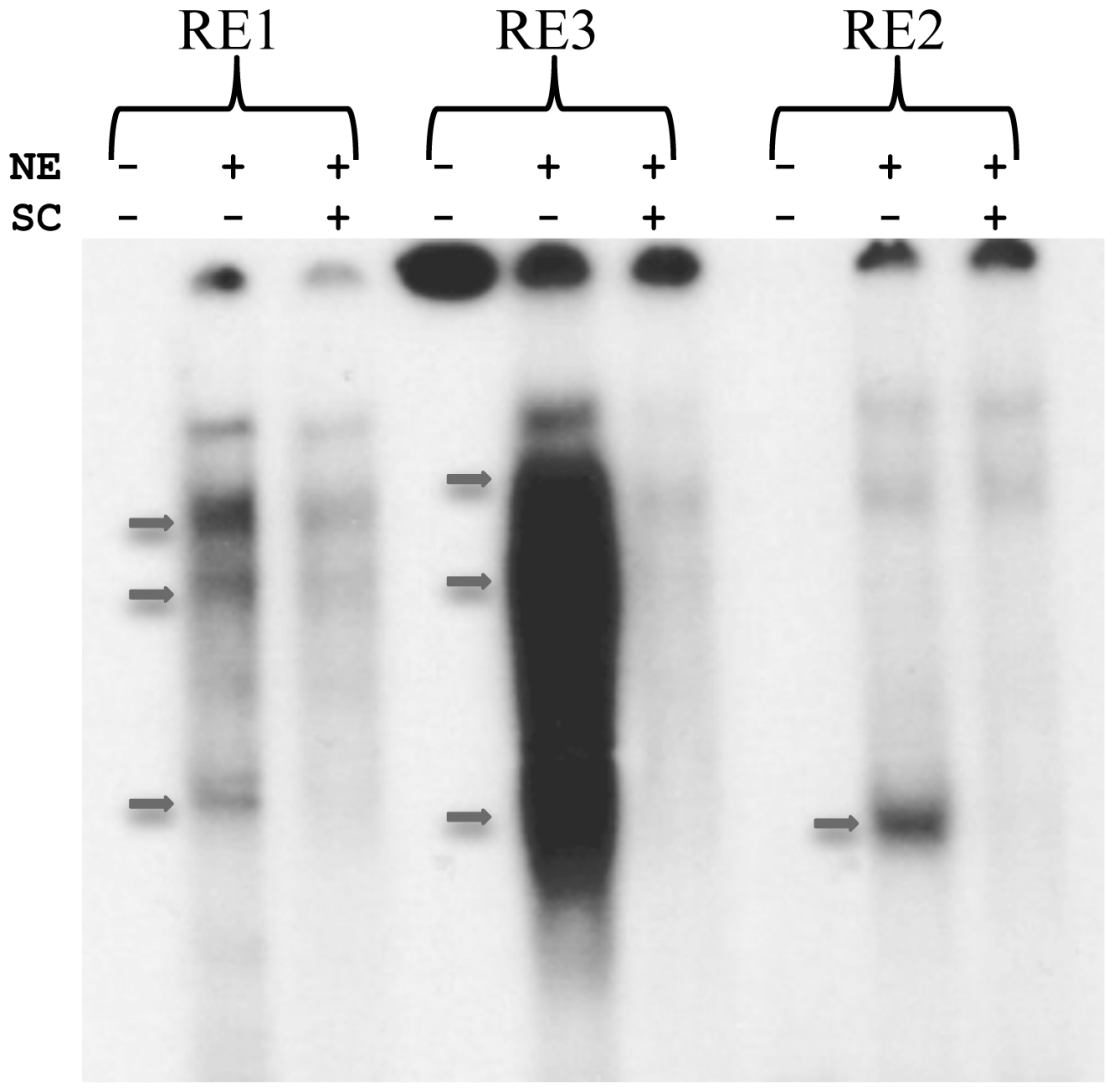
Specific binding of embryonic nuclear proteins to elements RE1, RE2 and RE3. The DNA binding specificity of transcription factors to the elements RE1, RE2 and RE3 was determined by electrophoretic mobility shift assays. NE: Nuclear Extract; SC: Specific Competitor. (−) and (+) denote omission and addition of nuclear extract or specific competitor to each reaction respectively. The arrows point to protein: DNA complexes, which are not formed in the presence of specific competitor.

The nucleotide sequence of module *a*, was searched for transcription factor binding sites using MatInspector. This analysis produced a plethora of putative elements and we set out to test some of them within the sub-region from −452 to −333, which seems to contain significant cis-acting elements as our in vivo expression data suggest. Thus, three such elements, RE1 at −453, RE2 at −432 and RE3 at −377, were used as radioactive labeled probes to detect DNA binding proteins in embryonic (blastula stage) nuclear extracts. The EMSA experiment presented in figure 9 indicates that all three elements are specifically recognized by sea urchin transcription factors included into the nuclear extract, judged by the addition of unlabeled probes as specific competitors. RE1 and RE3 binding results in the formation of more than one specific complex, whereas RE2 seems to form a single complex.

### Site-specific mutations into module *a*

**Figure 10:**
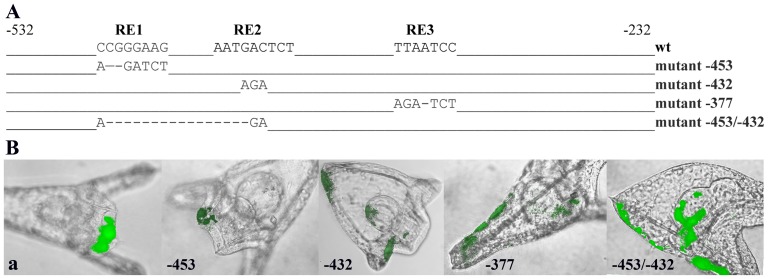
Spatial expression patterns of site-specific mutations into module *a*. **A**: Graphic presentation of the wt and mutant sequences of the three elements within module *a*. The top line depicts the 320 bp region of the wt module *a*, and the sequences that correspond to the sites RE1 (−453), RE2 (−432) and RE3 (−377) and their respective position. Each additional line shows the nucleotides that substitute for the wt sequence at each site. The bottom line depicts the double mutation, which deletes also the intervening sequences between RE1 and RE2. **B**: Composite pictures of embryos expressing GFP resulting from injection of wt and mutant module *a*. The wt module *a* and mutation −453 show GFP expression specifically in the ciliary band, while mutation −432 shows expression in ciliary band, endoderm and aboral ectoderm. Mutation −377 shows GFP expression in aboral ectoderm and the double mutation −453/−432 in aboral ectoderm, endoderm and ciliary band. All embryos were photographed at pluteus stage.

Based on the EMSA results we set out to investigate the role of the three cis-acting elements, using inverse PCR with substrate module *a* fused to the GFP cassette and substituting the presumed core nucleotides of each binding site with the recognition sequence of the restriction site BglII (5′agatct). We created also the double mutant (−453/−432), which in addition to the two elements RE1 and RE2 lacks the 20 bp of the intervening sequence (Fig. 10A). Mutation of the RE1 site (−453) shows no ectopic expression (Fig. 10B), but a considerable decrease in the number of fluorescent embryos (Table 1). Furthermore, the fluorescence exhibited by these embryos was barely noticeable over the detection limit of the microscope. Thus, we assign to the RE1 element, a positive role in the regulation of the *PlCoup-TF* gene. On the other hand, mutation of the RE2 site (−432) results in a two and a half fold increase of endodermal and a six fold increase in aboral ectoderm expression, with only a small decrease in ciliary band expression (Table 1). In addition, RE2 injected embryos exhibit normal levels of fluorescence. The expression of the mutant RE2 site is in agreement with the D1 and D-a2 deletions (Table 1), suggesting that RE2 is a negative regulatory element that is essential for the suppression of *PlCoup-TF* in the endodermal and aboral ectoderm territories of the pluteus embryo. Mutation of the RE3 site (−377) has no effect on ciliary band or endoderm expression (Fig. 10B), but results in a four and a half fold increase in aboral ectoderm expression, in accordance to the D2 deletion (Table 1). These results suggest that RE3 is a negative regulatory element, which suppresses *PlCoup-TF* expression specifically in the aboral ectoderm. Mutation of both RE1 and RE2 elements, results in a small percentage of fluorescent embryos as well as faint levels of fluorescence in all expressing territories. Moreover, the double mutation leads to a complete loss of spatial expression preference of the reporter gene. It is obvious that the double mutation shows the collective effects of the individually mutated RE1 and RE2 elements (Table 1). From the site-specific mutagenesis results, we conclude that the combined effects of these three upstream elements reproduce the regulatory capacity of the entire module *a*, and suffice for the correct quantitative and spatial expression of *PlCoup-TF* in the pluteus larva.

## Discussion

The herein study focuses on the regulation of the *PlCoup-TF* gene at later embryonic stages, where its expression is mainly restricted to the ciliary band of the pluteus as demonstrated by in situ hybridization (figure 1). This restriction follows a broader zygotic activation of the gene at earlier stages (blastula through gastrula), subsequent to the turnover of the maternal RNA. Thus, our study does not take into account plausible additional regulatory elements responsible for its early activation, but rather relates to the mechanism that sustains its quantitative and spatial mode of expression in the pluteus ciliary band.

Coup-TF is an essential transcription factor in early development. Knockouts of ortholog genes in various animals, result in lethal phenotypes. In *Paracentrotus lividus*, injections of PlCoup-TF morpholino antisense oligonucleotides into fertilized eggs lead to developmental arrest at the blastula stage, with embryos that lack gut formation and show diminished spiculogenesis (unpublished data from this laboratory). Thus, the store of maternal RNA and protein seems to be adequate for the very early embryonic regulatory functions, but later on, when maternal Coup-TF transcripts and protein are turned over, the embryo relies greatly on newly synthesized zygotic transcripts. *Coup-TF's* embryonic spatial expression profile (oral ectoderm, ciliary band) and its restricted expression in neuronal cell types of the feeding larvae (unpublished data from this laboratory) are indicative of its role in sea urchin neurogenesis, similar to a variety of other organisms [13]–[15]. The present study elucidates the mechanism of *PlCoup-TF's* late embryonic regulation, as a first step in placing this transcription factor within the Gene Regulatory Network of the sea urchin oral ectoderm and ciliary band.

### The *PlCoup-TF* gene structure

Although the isolated overlapping P.lividus genomic clones span 33.5 kb of genomic sequences, they do not include the entire gene, since the last exon is not present. Presumably, the last intron of the gene is extremely long, as also seems to be the case for the *SpCoup-TF* gene. The scaffold containing the *SpCoup-TF* gene, assembled by the genome project of *Strongylocentrotus purpuratus*, also lacks the fourth exon.

We focused on the study of the 1930 bp upstream fragment contained in the cloned insert of phage “*Φ*”, mindful that additional regulatory regions may lie further upstream or downstream of the chosen area. Two of the multiple transcription initiation sites that we found, spaced 15 bp apart, seem to account for the majority of the 5′-ends of the mature transcripts. The nucleotide sequence in the upstream vicinity of the +1 implies that the promoter of the gene is TATA-less. In addition, no other obvious promoter elements are recognized with the exception of a putative CCAAT element at position −65 (figure 3). While other unidentified promoter elements possibly exist, the proximal upstream promoter of *PlCoup-TF*, as revealed by our deletion analysis, is not capable of spatial regulation of the gene. This is evident from embryos expressing deletion −232 (figure 4), which exhibit GFP expression to the same extent in all observed embryonic territories. Thus, considering these 232 bp as the proximal promoter, they provide positive inputs to the *Endo16* basal promoter without any territorial restriction. Further proximal promoter inputs downstream of the +1 site are also possible, but they are not included in the herein analysis.

### Identification of a single regulatory module

Since the endogenous *PlCoup-TF* gene is primarily expressed in the ciliary band of the pluteus stage, groups of embryos injected by a given construct and exhibiting high percentage of fluorescence primarily in the ciliary band, would indicate that the construct contains the necessary *cis* acting elements for correct embryonic regulation of *PlCoup-TF* at this late developmental stage. It is also expected that, in this type of analysis, any given tested sequence will be expressed in more cell types than in those in which its regulatory milieu is capable of driving it. The latter, results from the fact that the injected DNA is integrated randomly into the genome [31], becoming thus influenced by surrounding *cis* acting elements.

Stepwise 5′ deletions of the 1930 bp upstream region and internal segments thereof, driving the GFP expression cassette, demonstrate that a single 320 bp module (module *a*, −532 to −232) is necessary and sufficient to recapitulate the endogenous *PlCoup-TF* expression in the pluteus larva (figures 4 and 5). Segments b and c, upstream of −532, exhibit reduced percentage of fluorescent embryos and loss of ciliary band specificity, whereas further upstream segments (d–f) are unable to drive the GFP expression cassette and thus, considered devoid of relevant cis-acting elements. Downstream though of module *a*, within the region from −232 to +1, positive acting element(s) must account for the observed expression of GFP in all embryonic territories (Table 1). Such elements, comprising perhaps the *PlCoup-TF's* basal promoter, do not confer any restriction to spatial expression. It is evident therefore, as the −232 and −532 deletion data suggest, that module *a* must be comprised of negative regulatory elements that restrict expression of the gene to the ciliary band. Module *a* must also contain positive acting elements, recognized by ciliary band expressed factors, since it is able by itself to drive the expression of the GFP cassette (Fig. 5B) in this territory. Module *a* thus, is necessary and sufficient to provide proper spatial and quantitative regulatory inputs to the *PlCoup-TF* gene at pluteus stage.

Comparison of *PlCoup-TF's* upstream sequence with the upstream sequence of the ortholog *SpCoup-TF* gene from *Strongylocentrotus purpuratus* should permit the identification of evolutionary conserved regulatory sites [37]. Such homologous segments are generally considered to contain important regulatory elements, conserved because of their functionality. Interestingly though, none of the identified conserved segments are found within module *a*. It is conceivable either that the conserved upstream sequences are not important for the regulation of the *PlCoup-TF* gene, or that they may be necessary for some other aspect of the gene's regulation, which is not revealed by the herein analysis. It is possible that similar sequences to module *a* maybe found in further upstream or downstream regions of the *SpCoup-TF* gene, not included in this comparison, which would suggest similar regulatory inputs for temporal and spatial embryonic regulation of the two genes. On the other hand, our results may indicate that the two genes use different regulatory factors to attain the same expression pattern in the pluteus embryo.

### Regulatory sites comprising module *a*

A minimal 120 bp segment within module *a*, was shown to confer ciliary band specific expression to the *PlCoup-TF* gene. An *in silico* analysis of this segment (not shown) revealed a wealth of putative transcription factor binding consensus sequences. The position of three elements, RE1, RE2 and RE3, identified through electrophoretic mobility shift assays correlates with the functional deletion analysis of small segments within module *a*, suggesting that the three elements may be involved in functional DNA-protein interactions. This argument does not exclude the possibility that other functional cis- acting elements might exist within the minimal 120 bp segment. The assumption we took is that these three elements, to be real regulatory sites, should fulfill the following criteria, stemming from the functional deletion analysis, i.e. they should provide both positive and negative inputs to the *PlCoup-TF* gene. Thus, site-specific mutagenesis of the three discrete elements was undertaken to investigate their specific role in the context of the entire module *a*, rather than the minimal 120 bp segment, to provide a more significant functional consequence of each mutation.

### Functional analysis of the cis- acting elements RE1, RE2 and RE3

Mutation of the RE1 site implies that the corresponding binding factor(s) provides a positive input into the *PlCoup-TF* gene. The observed weak fluorescent signal was mostly associated with cells of the ciliary band, suggesting that the RE1 site does not provide any spatial regulatory clues to the gene. Thus, it is through RE1 that *PlCoup-TF* receives the major positive input at pluteus stage embryos. The weak fluorescence observed in some embryos, vs. total lack of expression, is perhaps indicative of additional minor positive inputs within module *a*. The mutated sequence of RE1 (GGAAG, Fig. 10A) corresponds to the consensus binding sequence of Ets transcription factors. An identical element is found in the CyIIa gene promoter, responding also to Ets factors [38]. Two members of the sea urchin *S.purpuratus* Ets gene family, *SpElk* and *SpPea*, are expressed at the pluteus stage embryo and predominantly in the oral ectoderm [39]. As in *S.purpuratus*, the *P.lividus* ortholog genes *PlElk* and *PlPea* were shown by *in situ* hybridization to be expressed in the ciliary band of the pluteus stage embryo (unpublished results from this laboratory). Thus, one or both of these Ets factors could account for the positive regulation of the *PlCoup-TF* gene. It is interesting to note that the mouse *Coup-TFI* gene, NR2F1, is also positively regulated by Ets1, a member of the family [25]. This is not surprising, since the Ets family of transcription factors is very conserved in the animal kingdom and involved in a plethora of developmental processes.

The analysis of the RE2 and RE3 mutants suggests that these cis-acting elements are recognized by repressors, which act at embryonic territories other than the ciliary band and oral ectoderm to suppress the expression of *PlCoup-TF*. The unknown repressor that binds to the RE2 element seems to extend its influence in all such embryonic territories, i.e. endoderm, mesoderm and aboral ectoderm, since mutation of RE2 results in total loss of spatial restriction of the GFP cassette expression (Table 1). The double RE1/RE2 mutant shows an additive effect of the individual mutations, i.e. very few fluorescent embryos, weak GFP expression and total loss of spatial restriction. Thus the combinatorial effect of the RE1 and RE2 elements seems to account for most of the regulatory inputs for the proper quantitative and spatial regulation of the *PlCoup-TF* gene at pluteus stage.

Additional negative inputs are exerted to the gene through the RE3 element. These inputs though, in contrast to the RE2 element, seem to be specific to the embryonic aboral ectoderm of the pluteus, since the RE3 mutant GFP cassette looses spatial restriction only to this territory. Thus, the corresponding RE3 binding factor(s) should be a repressor expressed specifically in the aboral ectoderm of the embryo, where, in addition to the negative function of the RE2 binding factor, it silences further the expression of the PlCoup-TF gene.

The RE2 and RE3 sequences and the corresponding nuclear protein binding assays presented herein, do not give us any clues as to the identity of the two repressors. Future experiments will be designed to identify these factors, taking advantage of the significant progress made so far in the characterization of the gene regulatory networks that control the specification of the late embryonic territories [40]–[42].
